# Modifiable and Nonmodifiable Risk Factors for Breast Cancer: A Comprehensive Scoping Review

**DOI:** 10.1155/ijbc/8689986

**Published:** 2026-04-07

**Authors:** Zohre Momenimovahed, Zahra Shahabinia, Leila Allahqoli, Hamid Salehiniya

**Affiliations:** ^1^ Department of Reproductive Health, Qom University of Medical Sciences, Qom, Iran, muq.ac.ir; ^2^ Birjand University of Medical Sciences, Birjand, Iran, bums.ac.ir; ^3^ Faculty of Health Sciences, Cyprus International University, Nicosia, Cyprus, ciu.edu.tr; ^4^ Department of Epidemiology and Biostatistics, School of Health, Social Determinants of Health Research Center, Birjand University of Medical Sciences, Birjand, Iran, bums.ac.ir

**Keywords:** breast cancer, epidemiology, review, risk factor

## Abstract

**Background and Objectives:**

This scoping review focuses on gathering and examining the literature regarding modifiable and nonmodifiable risk factors for breast cancer.

**Materials and Methods:**

A comprehensive search was conducted in the PubMed/MEDLINE, Scopus, and Web of Science Core Collection databases with the keywords “breast cancer”, “risk”, and “risk factor” and a combination of these words to find quality articles published from 2010 to January 2025.

**Results:**

In total, 169 articles published in English were included in the study. The findings of this study revealed that family history, genetics, blood group, race, previous history of breast cancer, sex hormones, and breast density are nonmodifiable risk factors for this cancer. Smoking, alcohol, coffee and tea consumption, a low level of physical activity, dietary regimens, hormone replacement therapy, contraceptive pills, reproductive factors, no breastfeeding, metabolic syndrome, obesity, diabetes, some medications, work status, and other diseases are modifiable risk factors that play a role in the occurrence of breast cancer.

**Discussion:**

Lifestyle modifications such as improving one′s diet, engaging in regular physical activity, avoiding smoking/passive smoking, limiting alcohol consumption, maintaining a healthy weight after menopause, optimizing long‐term nonhormonal fertility choices, avoiding medically unnecessary hormone increases, and performing genetic screening when necessary can help in the prevention of breast cancer.

## 1. Introduction

Breast cancer ranks among the most common cancers affecting women globally, contributing to substantial cancer‐related mortality. In 2019, there were 1,977,212 new female patients with breast cancer globally [1]. Numerous risk factors play a role in breast cancer development. While the disease is present globally, its incidence, mortality, and survival rates differ significantly across various regions and are potentially influenced by different risk factors [2]. Understanding the risk factors linked to breast cancer is essential for developing efficient prevention tactics and enhancing early detection techniques [3]. Breast cancer risk factors can be divided into two primary categories: modifiable factors and nonmodifiable factors [4].

Nonmodifiable risk factors, such as characteristics, such as age, genetic predispositions (especially mutations in the BRCA1 and BRCA2 genes), and a personal or family history of breast cancer, refer to aspects that cannot be altered or affected by personal behavior or lifestyle decisions [5]. Conversely, modifiable risk factors, including excessive body weight, limited physical exercise, alcohol intake, and cigarette smoking, are aspects that people can affect by altering their lifestyle choices [6, 7].

Review articles serve as a key resource for program development and policymaking, offering researchers a comprehensive perspective on the numerous facets of a phenomenon [8, 9]. This scoping review is aimed at examining the worldwide risk factors that lead to the development of breast cancer by synthesizing and analyzing data from various population‐based studies carried out in different areas globally.

## 2. Methods

Published articles about breast cancer risk factors were searched for a scoping review.

### 2.1. Search Strategy

In line with the PRISMA guidelines [10], this research was carried out to address the following question: What are the modifiable and nonmodifiable risk factors for breast cancer worldwide? To address the study question, comprehensive searches were conducted in PubMed/MEDLINE, Scopus, and the Web of Science Core Collection (SCI‐EXPANDED, SSCI, and ESCI) from 2010 to January 2025. Keywords such as “breast cancer”, “risk”, and “risk factor” were utilized to search for relevant articles. To enhance the search′s thoroughness, two researchers also conducted a manual search of legitimate journals, which was succeeded by a manual search of the references cited in the obtained full‐text articles. All the obtained articles were loaded into EndNote 21. The search approach was adjusted repeatedly to increase the sensitivity and specificity of the articles.

### 2.2. Inclusion Criteria

We included original studies published in English that contained keywords in the title, abstract, or text.

### 2.3. Exclusion Criteria

The studies excluded commentaries, editorials, systematic reviews, conference abstracts, opinion statements, practice guidelines, case series or case reports, inability to access the full text of the article, and duplicate data present in multiple articles.

### 2.4. Data Extraction

The articles were located by one of the researchers (H.S.) and subsequently imported into EndNote 21. The criteria for including and excluding studies were independently reviewed by two researchers (Z.M. and H.S.) via the titles and abstracts of the articles. At this point, the articles that failed to meet the inclusion criteria were discarded, and subsequently, the complete texts of all the remaining articles that fulfilled the inclusion criteria were examined and evaluated. By closely examining each article, the results and conclusions were derived. Conflicts in every stage were settled by two referees via conversation.

## 3. Results

### 3.1. Selection of Studies

**Figure 1:**
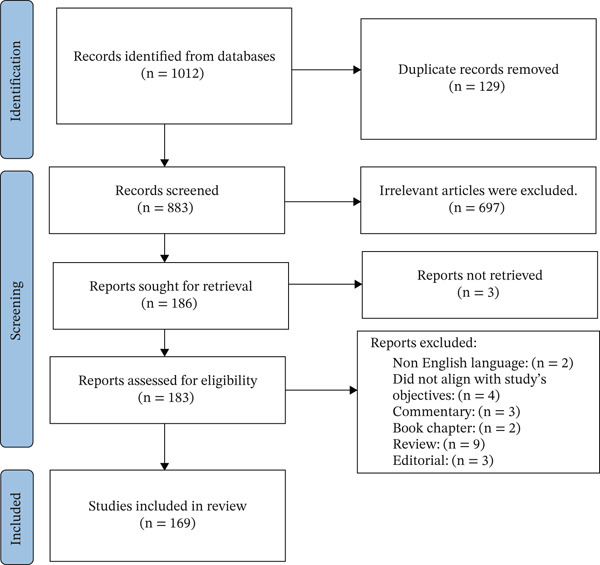
PRISMA flow diagram.

**Figure 2:**
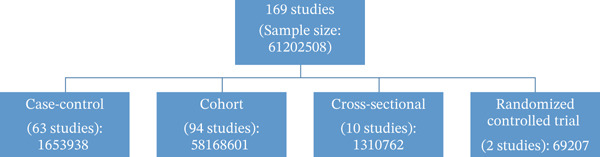
Number of included studies.

Upon exploration of various databases, a total of 1012 articles were obtained and input into EndNote 21. Among these, 129 articles were identified as duplicates. A total of 697 studies were omitted after their titles and abstracts were examined. Twenty‐three articles were excluded for the following reasons: non‐English language: 2; did not align with the study′s objective: 4; commentary: 3; book chapter: 2; review: 9; and editorial: 3. Nine articles were added from the manual search. Ultimately, 169 research articles were included in the systematic review (Figure 1). Figure 2 illustrates the types of studies included, along with their corresponding numbers and sample sizes.

### 3.2. Risk Factors (Table 1)

**Table 1:** Modifiable and nonmodifiable breast cancer risk factors.

Risk factor		Protective	Predisposing	Controversial
Nonmodifiable factors	Family history		✓	
	Genetics		✓	
	Blood group			✓
	Race		✓	
	Previous history of breast cancer		✓	
	Sex hormones		✓	
	Breast density		✓	
Modifiable factors	Smoking			✓
	Alcohol		✓	
	Coffee and tea consumption			✓
	Physical activity	✓		
	Dietary regimen			✓
	Hormone replacement therapy		✓	
	Contraceptive pills			✓
	Reproductive factors	✓		
	Breastfeeding	✓		
	Metabolic syndrome		✓	
	Obesity		✓	
	Diabetes			✓
	Medication			✓
	Work status			✓

#### 3.2.1. Nonmodifiable Factors

##### 3.2.1.1. Family History

Studies have shown that a family history increases the risk of breast cancer [11–14]. A first‐degree family history is associated with a greater risk of developing breast cancer than no first‐degree family history [12, 15]. Studies have indicated that the association of breast cancer in women with a first‐ and second‐degree family history is stronger than that in those with no family history [15, 16]. According to the results of one study, in women who have two or more relatives with breast cancer, the risk of breast cancer increases by up to 2.5 times (95% confidence interval [CI]: 1.83–3.47) [17].

##### 3.2.1.2. Genetics

One of the most common mutations that increases the risk of breast cancer is the BRCA1 and BRCA2 mutations. The incidence of breast cancer in early adulthood increased rapidly until the age of 30–40 years for BRCA1 carriers and until the age of 40–50 years for BRCA2 carriers; then, the incidence of breast cancer remained similar and stable (20–30 per 1000 individuals per year) until the age of 80 years [18]. For breast cancer patients, the cumulative risk 20 years after the diagnosis of breast cancer was 40% for BRCA1 carriers and 26% for BRCA2 carriers. The risk of developing breast cancer increases with the number of first‐ and second‐degree relatives diagnosed with breast cancer for both BRCA1 and BRCA2 [18]. Specific genetic polymorphisms in DNA damage repair genes are also significantly associated with the risk of breast cancer [19]. Individuals within the highest percentile of polygenic risk scores (PRSs) exhibited a lifetime breast cancer risk of 32.6%. When benchmarked against the median‐risk quintile, subjects in the 99th percentile of risk for the subtype‐specific PRS313 demonstrated a 4.37‐fold increased risk for estrogen receptor (ER)–positive (ER+) breast cancer and a 2.78‐fold increased risk for ER‐negative breast cancer. Conversely, those in the first percentile showed a substantially reduced risk, with hazard ratios (HRs) of 0.16 and 0.27 for ER+ and ER‐negative disease, respectively [20].

##### 3.2.1.3. Blood Group

A study of 442 patients in 2016 revealed that patients with Blood Groups A, B, and AB were more likely to develop aggressive breast cancer [21]. A study conducted by Mjali et al. reported that blood group was significantly associated with histopathological status (*p* < 0.001) [22]. The findings of a case–control study indicate that individuals with Blood Group A have a significantly greater risk of developing breast cancer (relative risk [RR] = 3.447). Similarly, individuals with Blood Group AB also had a greater risk (RR = 3.659). In contrast, there was no significant difference in the risk of cancer between individuals with Blood Groups B and O [23]. The findings of the study by Kumari et al. revealed a strong association between blood type and the risk of breast cancer, with Blood Type A exhibiting the highest frequency of breast cancer cases, followed by Blood Types B and O [24]. However, many studies have not reported a correlation between blood type and the risk of breast cancer [25–27]. These studies indicated that the type, grade, stage, and hormonal status of breast cancer do not have a significant relationship with the ABO blood group [28] and that the blood type or Rh factor is not a prognostic indicator of survival or progression‐free survival [29]. Differences in study design, sample size, demographic diversity, and various modulating variables, such as hormonal factors, as well as certain biological mechanisms, contribute to the variability in research results. Blood group antigens may influence cell adhesion, the immune response, or inflammation [30, 31]. Additionally, some coagulation factors, such as von Willebrand factor, may play a role in metastasis [32]. These mechanisms are less studied and therefore may explain the differences in results.

##### 3.2.1.4. Race

Inequalities in the incidence of breast cancer based on race/ethnicity still exist, although there are significant differences among subgroups of women [33, 34]. Compared with non‐Hispanic White women, American Indian/Alaskan Native, Asian Indian/Pakistani, Black, Filipino, Hawaiian, Mexican, Puerto Rican, and Samoan women had 1.3–7.1‐fold greater odds of presenting with Stage IV breast cancer [35]. The results of one study revealed that among American women diagnosed with invasive breast cancer, the likelihood of being diagnosed at early stages and survival after a Stage I diagnosis varies on the basis of race and ethnicity [36]. Compared with non‐Hispanic White women diagnosed with Stage I breast cancer (50.8%), Japanese women (56.1%) were more likely, and Black women (37.0%) were less likely to be diagnosed with breast cancer [36]. African American and American Indian/Alaskan Native women have the highest risk of developing Stage IV triple‐negative breast cancer [37]. Non‐Hispanic Black, Asian American/Pacific Islander, Native American, and Hispanic women are more likely than non‐Hispanic White women to have invasive breast cancer at younger ages and at more advanced stages, as well as higher breast cancer mortality rates at younger ages [38]. Among women under 45 years of age, the incidence of breast cancer was highest among Black women. Compared with White and Asian or Pacific Islander women, Black, American Indian or Alaskan Native, and Hispanic women accounted for a greater proportion of diagnosed cases at later stages. Compared with other racial/ethnic groups, Black women had a greater proportion of Grade III–IV tumors. Among all age groups, the incidence of triple‐negative breast cancer among Black women is significantly greater than that among women of other races/ethnicities, and this disparity increases with age [39].

##### 3.2.1.5. Previous History of Breast Cancer

The risk of a second primary breast cancer is increased in women with a prior history of breast cancer [40–42]. After adjusting for age, country of residence, and cancer treatment, previous right breast cancer was recognized as a significant risk factor for left breast cancer in BRCA1 or BRCA2 mutation carriers [36]. The findings showed that breast cancer survivors had a 16% increased risk of developing subsequent cancers. This risk increased with longer follow‐up periods, with standardized incidence ratio (SIR) values of 1.04, 1.22, and 1.31 for follow‐up periods of 12–59 months, 60–119 months, and 120 months or more, respectively [41]. The increase in risk was primarily observed, especially in early‐onset cancer (28, 29), in primary HR‐negative cancers [41, 42], and among racial/ethnic minorities [41]. According to the findings of the Sung et al. study, breast cancer survivors with HR‐positive cancers were 20% more at risk of developing new cancers than the general population (SIR = 1.20), whereas those with HR‐negative cancers had a 44% increased risk (SIR = 1.44) [42].

##### 3.2.1.6. Sex Hormones

Research has revealed that early menarche [43–50] and late menopause [50] are associated with an increased risk of breast cancer. Day et al. confirmed a causal relationship between the delay in the natural age of menopause and an increased risk of developing breast cancer, estimating that for each year of delayed menopause, there is a 6% increase in risk [51]. However, El Sharif and Khatib reported that menarche after the age of 13 significantly doubles the risk of breast cancer (adjusted odds ratio [aOR] = 2.03; 95% CI: 1.21–4.37) [52]. Some studies have shown that there is a relationship between the age of menarche and the risk of certain specific types of breast cancer. According to the Phipps et al. study, the age at menarche and menopause is associated with the risk of ER+ but does not correlate with triple‐negative breast cancer [53]. Another study showed that younger age at menarche is associated with an increased risk of luminal breast cancer [45]. Minami demonstrated that older age at menarche is inversely related to more advanced tumors [46]. Another study in 2013 reported that age at menarche, parity, and the number of full‐term pregnancies are associated with the risk of ER+, triple‐negative, and HER2‐overexpressing breast cancers [54], although some studies did not confirm these findings. Age at menarche is not significantly correlated with breast cancer risk or type [55]. In addition to age at menarche, the length and pattern of the menstrual cycle have also been studied. Findings from one study indicated that, in postmenopausal patients, shorter menstrual cycles (≤ 30 years) were clearly reduced in HR‐positive patients (odds ratio [OR] 0.397; 95% CI: 0.22–0.71), whereas no similar pattern was found in other molecular subgroups [56]. In individuals with late menarche, having a regular menstrual cycle decreases the risk of breast cancer [57].

##### 3.2.1.7. Breast Density

Breast density in mammography [58–61], especially in older and postmenopausal women [60], increases the risk of breast cancer. Increased breast density is associated with an increased risk of breast cancer in pre‐ and postmenopausal women across all BMI categories (premenopausal women: adjusted risk ratio [aRR] = 2.36; 95% CI: 2.24–2.49; postmenopausal women: aRR = 2.91; 95% CI: 2.78–3.04) [59]. The results of a population‐based screening revealed that the absolute risk of breast cancer after the age of 50 years was 6.2% for the low‐density group and 14.7% for the high‐density group [61].

#### 3.2.2. Modifiable Factors

##### 3.2.2.1. Smoking

Many studies have been conducted on the relationship between smoking and breast cancer. Some studies have indicated that smoking increases the risk of breast cancer [11], particularly luminal A, luminal B, and HER2‐negative breast cancer [62]. Menopausal status significantly affects the relationship between smoking and the risk of breast cancer. The aOR for premenopausal women, especially those who have smoked for 30 years or more, was 1.75, indicating a significant increase in risk (aOR = 1.75; 95% CI: 1.04–2.94). In contrast, in postmenopausal women, smoking is associated with a lower risk of breast cancer (aOR = 0.69; 95% CI: 0.51–0.93) [63]. The risk of breast cancer also increases with exposure to secondhand smoke. According to the results of a case–control study, a significant increasing trend in ORs was observed with the number of cigarettes smoked daily by husbands, years of passive smoking by husbands, and pack‐years of smoking by husbands [64]. Dossus et al. reported that current, former, and passive smokers are at risk for breast cancer [65]. However, some studies have shown no association between smoking and the risk of breast cancer [66].

##### 3.2.2.2. Alcohol

Many studies have shown that alcohol increases the risk of breast cancer [64, 67–70]. Compared with not drinking alcohol, consuming more than six glasses increases the risk of luminal A breast cancer [62]. Consuming alcohol before the age of 15 increases the risk of breast cancer by up to two times (OR = 1.98; 95% CI: 1.06–3.69) [71]. Alcohol may increase estrogen levels, which is a known risk factor for breast cancer [72]. The results of a study among postmenopausal women indicate that alcohol consumption is not associated with the risk of all types of breast cancer. On the basis of the findings of this study, alcohol consumption is associated with an increased risk of ER+, PR+, ER+/PR+, and mixed ductal/lobular cancer [73].

##### 3.2.2.3. Coffee and Tea Consumption

The results of a case–control study indicate that high daily coffee consumption is associated with a reduced risk of ER‐negative breast cancer among postmenopausal women (OR > 5 cups/day compared with OR ≤ 1 cup/day: 0.43; 95% CI: 0.25‐0.72; *p* trend = 0.0003) [74]. This finding was also confirmed in a study by Lowcock et al. [75]. An examination of 42,099 women in Sudan also revealed that coffee and caffeine consumption reduce the risk of ER+/PR− cancer (RR 0.87; 95% CI: 0.76–1.00 for three to four cups; RR 0.81; 95*%*CI = 0.70–0.94 for ≥ 5 cups) [76]. Among postmenopausal women, more than one cup of coffee per day was associated with a lower incidence of breast cancer (HR = 0.44; 95% CI: 0.21–0.92) in a fully adjusted model than women who consumed one cup of coffee or less per day [77]. Other studies have shown that coffee is not associated with an increased risk of breast cancer [78–82] and that there is no relationship between caffeinated or decaffeinated coffee and breast cancer [81].

Research has shown that tea consumption is associated with an increased risk of ER+/PR+ breast cancer [76]. Other studies have indicated that there is no relationship between tea consumption and the risk of breast cancer [78, 79, 82, 83]. A Mendelian randomization study revealed that there is no causal relationship between tea consumption and the risk of breast cancer, and the slight increase in risk for each additional cup is not statistically significant [84].

##### 3.2.2.4. Physical Activity

Physical activity is associated with a reduced risk of breast cancer [85–89]. Compared with women who are physically inactive, women with high levels of physical activity have a lower risk of breast cancer (OR = 0.55; 95% CI: 0.41–0.75). In premenopausal women, a reduction in risk is only observed in those with a normal weight (OR = 0.31; 95% CI: 0.13–0.75), whereas in postmenopausal women, a reduction in risk is observed in obese women (OR = 0.29; 95% CI: 0.12–0.66) [90]. Compared with physical activity in the lower quartile, physical activity in the upper quartile was associated with a greater risk of cancer both before and after menopause (RR = 0.75; 95% CI: 0.60–0.93) [87]. Ellingjord‐Dale et al. reported that physical activity (over 6 h/week compared with a lack of physical activity) is associated with a 16% reduction in the risk of luminal A breast cancer and other types of breast cancer (OR = 0.84; 95% CI: 0.70–1.02) [62]. Some studies have indicated that higher levels of physical activity at younger ages are inversely related to the risk of breast cancer and support the idea of early life as a window of susceptibility to breast cancer [91, 92]. On the basis of the findings of the Lin et al. study, women with higher levels of physical activity from menarche to their first childbirth showed a reduced risk of invasive breast cancer [57].

##### 3.2.2.5. Dietary Regimen

Compared with the lower quartile, adherence to a Western dietary pattern was associated with a greater risk of breast cancer in the upper quartile (OR for the top quartile vs.the bottom quartile = 1.46; 95% CI: 1.06–2.01), particularly in postmenopausal women (OR = 1.75; 95% CI: 1.14–2.67) [93]. In contrast, the Mediterranean diet was linked to a lower risk [93, 94]. Although the harmful effect of the Western pattern was similarly observed across all tumor subgroups, the protective effect of the Mediterranean pattern was stronger for triple‐negative tumors [93]. Adherence to the Mediterranean diet, excluding alcohol, was associated with a moderate reduction in breast cancer risk in postmenopausal women, and this association was stronger for receptor‐negative tumors [95]. However, Castelló et al. reported in a contradictory study that there is no association between the Mediterranean diet and breast cancer risk [96].

The consumption of high‐fat dairy products, red and processed meats, refined grains, sweets, calorie‐laden beverages, sauces, and industrial trans fats may be associated with an increased risk of breast cancer [96–98], whereas vegetables, fruits, and healthy dietary patterns are negatively correlated with the risk of breast cancer [97]. Conversely, a diet rich in fruits, vegetables, legumes, fatty fish, and vegetable oils is important for the prevention of all subtypes of breast cancer, especially triple‐negative tumors [93]. Accordingly, although an overall inverse relationship with breast cancer risk was observed for the consumption of cruciferous vegetables [99], higher intakes of soy milk and soy products are associated with a lower risk of breast cancer [85].

##### 3.2.2.6. Hormone Replacement Therapy

HRT increases the risk of breast cancer [52, 100], and this risk increases with increasing duration of use [100, 101], especially in women with dense breasts [100]. Estrogen therapy combined with progesterone is associated with an increased risk of invasive breast cancer (HR = 1.54; 95% CI: 1.44–1.64), and in those who have used it for more than 10 years, the risk of breast cancer doubles [102]. However, Cordina‐Duverger et al. suggested that for those who use a combination of estrogen and natural micronized progesterone, the risk of breast cancer does not increase. In users of combined estrogen and progesterone therapy containing synthetic progestin, the risk associated with progestin derived from progesterone is 1.57 (95% CI: 0.99–2.49), and that associated with testosterone is 3.35 (95% CI: 1.07–10.4) [101]. Compared with nonusers, new users (less than 5 years) and long‐term users (more than 5 years) of estrogen‐only and combined estrogen‐progestin therapy are associated with an increased risk of breast cancer [103]. Cordina‐Duverger et al. reported that tibolone is associated with an increased risk of breast cancer [101]. However, on the basis of findings from a cohort study, the use of estrogen therapy was not associated with an increased risk of breast cancer [102].

##### 3.2.2.7. Contraceptive Pills

The use of oral contraceptive pills is associated with an increased risk of breast cancer [52, 103–105]. On the basis of the findings of one study, the use of combined pills is associated with the risk of ER− and ER−/PR− but not with the risk of ER+ and ER+/PR+ [106]. Among women carrying the BRCA1 mutation, the use of combined contraceptive pills is significantly associated with an increased risk of breast cancer in those who began taking the pills before the age of 20 (OR = 1.45; 95% CI: 1.20–1.75; *p* = 0.0001), and this association between the use of contraceptive pills and breast cancer is limited to cases of cancer diagnosed before the age of 40 (OR = 1.40; 95% CI: 1.14–1.70; *p* = 0.001) [107]. The risk of early breast cancer increases by 11% for each year of contraceptive pill usage when the pills are taken before the age of 20 (OR = 1.11; 95% CI: 1.03–1.20; *p* = 0.008) [107]. Among all types of hormonal contraception methods, the risk of breast cancer is highest in the first 5 years of use (47). The risk of breast cancer disappears 10 years after the use of these pills is stopped [108]. Balekouzou et al. reported that the risk of breast cancer decreases with the use of hormonal contraceptive methods [109]. Other studies have shown that the use of contraceptive pills is not associated with the risk of various types of breast cancer [53, 110]. Previous long‐term use of estrogen‐only therapy and short‐term (under 5 years) use of estrogen and progesterone are not associated with increased risk [103].

##### 3.2.2.8. Reproductive Factors

Nulliparity is significantly associated with an increased risk of breast cancer [48, 52], and this risk decreases with increasing parity [49, 111, 112] and the number of full‐term births [109, 113]. Some studies have indicated that parity is associated with certain types of breast cancer [114]; on the basis of the results of one study, having full‐term births, the number of births, age, and the time elapsed since the last birth are associated with only types of ER+PR+ [110]. Studies have shown that nulliparity is associated with a reduced risk of triple‐negative breast cancer (HR = 0.61; 95% CI: 0.37–0.97) and an increased risk of ER+ breast cancer (HR = 1.35; 95% CI: 1.20–1.52) [53], and the risk of ER+ breast cancer decreases in parous women (vs. nulliparous, HR = 0.82; 95% CI: 0.77–0.88) [115]. Findings from one study indicated that high parity (more than or equal to three) without breastfeeding is associated with the risk of ER−PR− tumors (OR = 1.57; 95% CI: 1.10–2.24) [116]. Parous women are more likely to be diagnosed with triple‐negative breast cancer than with luminal A breast cancer (OR for parity = 1.38; 95% CI: 1.16–1.65, *p* = 0.0004; *p* for interaction with age = 0.076) [55]. Fortner et al. reported that, compared with nulliparous women, higher parity is inversely associated with luminal B breast cancer regardless of breastfeeding status (≥ 3 children: ever breastfed, 0.78; 95% CI: 0.62–0.98; never breastfed: 0.76; 95% CI: 0.58–1.00) [115]. Abortion is a risk factor for breast cancer in multiparous women [117].

While a younger age at the first full‐term pregnancy is associated with a reduced risk of breast cancer [43, 112], a long interval from menarche to first childbirth is associated with an increased risk of breast cancer [57, 65, 118]. For each year of delay in the first pregnancy, women had a 4% greater chance of being diagnosed with ER+ tumors (OR = 1.04; 95% CI: 1.01–1.08) [48], and first full‐term childbirth at older ages was associated with an increased risk of ER+PR+ tumors (≥ 35 vs. ≤ 19 years, HR = 1.47, 95% CI: 1.15–1.88; *p* trend < 0.001) [110]. The results of one study indicated that older age at first childbirth (HR = 1.15; 95% CI: 1.05–1.26, for each 5‐year increase in age) and lower parity are associated with an increased risk of luminal breast cancer [45]. Li et al.′s study in 2013 revealed that age at first live childbirth (*p* value for trend = 0.002) and the interval between menarche age and first childbirth age (*p* value for trend = 0.006) are inversely related to the risk of triple‐negative breast cancer but are not associated with the risks of ER+ and HER2 [54]. However, this finding was not confirmed in other studies. The results of one study indicated that age at first childbirth has no effect [119], and Abubakar et al. reported that older age at first childbirth is associated with a favorable prognosis [111].

##### 3.2.2.9. Breastfeeding

Breastfeeding [85, 111, 113, 115] and its longer duration [85, 86] are associated with a reduced risk of breast cancer. Compared with women with shorter breastfeeding periods, a longer duration is associated with a 93% reduction in the risk of breast cancer [86]. Among BRCA carriers, breastfeeding for at least 1 year was associated with a 32% reduction in breast cancer risk (OR = 0.68; 95% CI: 0.52–0.91; *p* = 0.008). Breastfeeding for 2 years or more reduces the risk even further (OR = 0.51; 95% CI: 0.35–0.74). In BRCA2 mutation carriers, there was no significant association between breastfeeding for at least 1 year and breast cancer risk (OR = 0.83; 95% CI: 0.53–1.31; *p* = 0.43) [120].

Many studies have investigated the protective effect of breastfeeding on specific types of breast cancer. Although the risk of ER− breast cancer decreases with breastfeeding, breastfeeding does not provide any other benefit for ER+ cancer patients [121]. Another study showed that while breastfeeding is associated with a reduced risk of certain types of HER2 and 5NP, it does not change the risk of other types [45]. Li et al. also reported that breastfeeding is inversely related to the risk of triple‐negative breast cancer but is not related to the risk of ER+ and HER2 [54], although this finding was not confirmed in other studies [53, 110].

##### 3.2.2.10. Metabolic Syndrome

Metabolic syndrome is associated with an increased risk of breast cancer [122–131], and this risk is significantly greater in postmenopausal women than in premenopausal women [123, 127, 128, 130, 132–136]. Having one of the risk factors for metabolic syndrome is associated with a 14% increase in breast cancer risk (HR = 1.14; 95% CI: 1.03–1.25), and as the number of risk factors increases, the risk of breast cancer also increases. Compared with individuals without risk factors, those with four risk factors for metabolic syndrome are associated with a 45% increase in breast cancer risk (HR = 1.45; 95% CI: 0.99, 2.13) [130]. In metabolic syndrome, obesity and dyslipidemia have the greatest adverse effects on postmenopausal breast cancer [135]; the number of components of metabolic syndrome affects breast cancer risk differently with respect to age. Findings from a study indicate that a greater number of components leads to an increased risk in older women (HR = 1.146; 95% CI: 1.123–1.170) and a decreased risk in younger women (HR = 0.915; 95% CI: 0.892–0.939) [136]. A study from Italy revealed that among the components of metabolic syndrome, only high blood glucose was significantly associated with an increased risk of breast cancer in all women (HR = 1.47; 95% CI: 1.13–1.91) and postmenopausal women (HR 1.89; 95% CI: 1.29–2.77) but not in premenopausal women (HR = 0.80; 95% CI: 0.52–1.22; *p* interaction = 0.004) [128]. Metabolic syndrome increases the risk of ER+ and PR+ breast cancer [124]. The results of another study indicated that the metabolic syndrome score is associated with a worse prognosis for patients with breast cancer [125] and an increased risk of breast cancer mortality [137].

##### 3.2.2.11. Obesity

Women with increased weight [69, 138] and obesity [71, 117, 138–141] are at risk for breast cancer. Grade 2 and 3 obesities are associated with advanced disease, larger tumor size, positive lymph nodes, and mortality following breast cancer [138]. However, the results of one study revealed that an increase in BMI increases the risk of less aggressive tumors (HR = per 5 kg/m^2^: 1.44; 95% CI: 1.10, 1.90; *p* = 0.009) [139].

The relationship between obesity and hormone receptor–positive breast tumors is strong [142]. While the risk of breast cancer increases with obesity in postmenopausal women [49, 140, 143], in premenopausal women, this risk decreases [140]. Weight gain in adulthood is associated with an increased risk of postmenopausal breast cancer, and for every 5 kg of weight gained from the age of 20, the risk increases by 6% (HR = 1.06; 95% CI: 1.01–1.11) [144]. Some studies have indicated that BMI at age 18 is definitely inversely related to breast cancer [142, 145].

In postmenopausal women with a normal BMI, relatively high levels of body fat were associated with an increased risk of invasive breast cancer and changes in circulating metabolic and inflammatory factors. Therefore, a normal BMI classification may be an inadequate proxy for breast cancer risk in postmenopausal women [146]. According to previous studies, total body fat percentage, android body fat, the android–gynoid ratio, and waist circumference are positively correlated [88]. Among women with a normal weight (18.5 ≤ BMI < 25), those with central obesity (WC > 88 cm) had a greater risk than women with a normal WC did (OR = 3.60; 95% CI: 1.47–8.79) [147]. Different fat distributions in adulthood are associated with varying risks of breast cancer [147]. Compared with stable weight, the risk of breast cancer was lower in postmenopausal women who experienced weight loss (HR = 0.88; 95% CI: 0.78–0.98; *p* = 0.02). Weight gain in these women was not associated with an increased risk (HR = 1.02; 95% CI: 0.93–1.11), but the risk of triple‐negative breast cancer increased (HR = 1.54; 95% CI: 1.16–2.05) [148].

##### 3.2.2.12. Diabetes

There is no association between Type 2 diabetes or GDM (gestational diabetes mellitus) and the risk of breast cancer [149–151]. According to the results of one study, the overall risk of breast cancer among women with and without previous GDM was similar, with an adjusted HR of 0.96 (aHR = 0.96; 95% CI: 0.83–1.12). There was no significant difference in the risk of breast cancer based on menopausal status. This study revealed no effect of the severity of insulin resistance or subsequent diabetes on the risk of breast cancer [152]. Compared with not having Type 2 diabetes, Type 2 diabetes treated with metformin is not associated with the overall risk of breast cancer (HR = 0.98; 95% CI: 0.83–1.15) but is linked to a reduced risk of both ER+ breast cancer (HR = 0.86; 95% CI: 0.70–1.05) and an increased risk of ER− (HR = 1.25; 95% CI: 0.84–1.88) and triple‐negative breast cancer (HR = 1.74; 95% CI: 1.06–2.83). Furthermore, an increase in metformin consumption is associated with a more significant reduction in the risk of ER+ breast cancer [150].

The occurrence of breast cancer in diabetic women varies with the type of medication used. Metformin reduces the risk of breast cancer [153–155], and the risk decreases further with increasing duration of use [153, 154]. This protective effect is enhanced in those who concurrently use statins [154]. Diabetic women who take medications other than metformin have a higher incidence of breast cancer (HR = 1.16; 95% CI: 0.93–1.45), whereas the incidence is lower in women who use metformin (HR = 0.75; 95% CI: 0.57–0.99). This association is observed in ER+ and PR+ cancers and in those negative for human epidermal growth factor receptor 2 [156]. Patients who are on metformin at the time of breast cancer diagnosis have a better prognosis if they are hormone receptor‐positive and HER2‐positive. According to the findings of this study, the absence of metformin use was associated with higher rates of mortality and metastasis [157]. However, this finding was not confirmed in other studies, and Lega et al. reported that there is no correlation between metformin use and the stage of breast cancer or tumor characteristics [158].

##### 3.2.2.13. Medication

Exposure to all antipsychotic medications was independently associated with a 35% increase in the risk of breast cancer (aHR = 1.35; 95% CI: 1.14–1.61) [159]. A greater risk was observed between the use of antipsychotic medications and the increased risk of breast cancer, with a stronger differential association with categories of antipsychotics that increase prolactin [159]. The results indicated that long‐term use of certain medications, such as valproic acid (OR = 0.58; 95% CI: 0.39–0.56), citalopram (OR = 0.58; 95% CI: 0.37–0.91), and sertraline (OR = 0.77; 95% CI: 0.61–0.91), was linked to a lower risk of breast cancer than lower doses. Additionally, short‐term use of fluvoxamine (OR = 0.82; 95% CI: 0.69–0.96), olanzapine (OR = 0.54; 95% CI: 0.33–0.89), risperidone (OR = 0.7; 95% CI: 0.51–0.98), and chlorpromazine (OR = 0.48; 95% CI: 0.25–0.90) was associated with a reduced risk. Importantly, this study did not find any evidence that the use of psychoactive medications increases the risk of breast cancer in these patients [160]. This finding was also confirmed in another study [161].

This study revealed a significant association between breast cancer risk and the expression of the SLC12A2 gene. Reduced expression of the SLC12A2 gene associated with antihypertensive medications was linked to a 16% increase in breast cancer risk (OR = 1.16; 95% CI: 1.06–1.28) [162]. A nationwide study revealed no significant associations between low‐dose aspirin, clopidogrel, or dipyridamole and breast cancer risk [163]. The results of a study regarding the association of NSAIDs with breast cancer risk indicated that women with benign breast disease who used NSAIDs had a lower risk of developing breast cancer (HR = 0.87; 95% CI: 0.78–0.97). This effect was consistent for both ER+ (HR = 0.85; 95% CI: 0.74–0.97) and ER‐negative (HR = 0.87; 95% CI: 0.59–1.29) breast cancer. Conversely, NSAID use was not significantly associated with breast cancer risk in women without benign breast disease [164]. A reduced risk of postmenopausal breast cancer is associated with current vitamin D supplementation (HR = 0.82; 95% CI: 0.69, 0.97) but not previous use (HR: 1.10; 95% CI: 0.92, 1.31) [165].

##### 3.2.2.14. Work Status

In working women, who are engaged in occupations with high exposure to carcinogens and endocrine disruptors [166], as well as women who work night shifts [167–171], particularly postmenopausal women [172], the risk of breast cancer increases. Night shifts increase the risk of breast cancer after prolonged exposure [173]. In nurses with permanent night shifts, in addition to rotating night and day shifts, breast cancer risk increases (OR = 2.9; 95% CI: 1.1–8.0) [174]. Findings from studies indicate that a panel of clock genes and circadian miRNAs may act as biomarkers for susceptibility to breast cancer among night shift workers [170, 171]. This study revealed significant differences in the expression of clock genes between breast cancer tissues and normal tissues. Specifically, genes such as BHLHE40, CIART, CLOCK, PDPK1, and TIMELESS are overexpressed, whereas HLF, NFIL3, NPAS3, PER1, PER3, SIM1, and TEF are underexpressed, which may contribute to the increased risk of breast cancer in these women [170].

Compared with women who have sleep durations of 8.9–6.1 h, women with shorter or longer sleep durations have an increased risk of breast cancer. Daytime napping is associated with a reduced risk of breast cancer among night shift workers (OR = 0.57; 95% CI: 0.36–0.90) [167]. The RR for breast cancer increases with the number of years worked on the night shift (*p* = 0.03) and the total number of shifts (*p* = 0.02), with a neutral risk for fewer than three night shifts per week [175]. Although some studies have shown that working night shifts is not associated with an increased risk of breast cancer [176, 177], the results of one study indicated that the risk of breast cancer related to night shift work was not correlated with the occupational exposure matrix or history of night shift work. Furthermore, risk was not associated with the frequency, duration, or cumulative amount of night shift work [176].

##### 3.2.2.15. Other Diseases

Periodontal disease is associated with an increased risk of breast cancer among postmenopausal women, particularly smokers who have quit smoking in the last 20 years [178]. According to the results of a case–control study, women diagnosed with periodontitis had two to three times greater odds of developing breast cancer than women without periodontitis, depending on the definition of periodontitis (*p* < 0.05) [179]. A study from Sweden revealed that 5.5% of individuals with periodontal disease and lost molars in the lower jaw had breast cancer, whereas 0.5% of individuals with periodontal disease but no lost molars in the lower jaw had breast cancer (*p* < 0.02). It seems that chronic periodontal disease, as indicated by lost molars, is statistically associated with breast cancer [180]. However, other studies have shown no association between periodontitis and breast cancer [181–183].

In a large observational study, no significant difference in the incidence of breast cancer was observed between women with endometriosis and those without endometriosis (2.4% vs. 2.5%) [184]. The results of a contradictory study indicated that endometriosis was associated with a significantly reduced risk of breast cancer (OR = 0.12) [185].

## 4. Discussion

This study identified modifiable and nonmodifiable risk factors for breast cancer via a comprehensive scoping review. The findings of this study revealed that family history, genetics, blood group, race, previous history of breast cancer, sex hormones, and breast density are nonmodifiable risk factors for this cancer. Smoking, alcohol, coffee and tea consumption, a low level of physical activity, dietary regimens, hormone replacement therapy, contraceptive pills, reproductive factors, no breastfeeding, metabolic syndrome, obesity, diabetes, some medications, work status, and other diseases are modifiable risk factors that play a role in the occurrence of breast cancer.

Nonmodifiable factors influence the occurrence of breast cancer through various mechanisms. Potent carcinogens in tobacco smoke, including polycyclic aromatic hydrocarbons (PAHs), aromatic amines, and nitrosamines, are implicated in the increased risk of smoking‐related breast cancer [186]. Research indicates that the consumption of cigarettes is linked to an increased risk of breast cancer through various biological pathways, including DNA damage and mutagenesis [187, 188], hormonal disruption [186], epigenetic alterations [189], chronic inflammation [190, 191], and metastasis promotion [192].

The way in which physical activity lowers the risk of breast cancer involves multiple biological mechanisms. Physical activity can cause hormone regulation and estrogen reduction [193], insulin and IGF‐1 pathway modulation [194], a reduction in body fat and adipokines [195], anti‐inflammatory effects [196], increased natural killer (NK) cell activity [197], a reduction in oxidative stress and DNA damage [198], and epigenetic regulation [199]. This protective effect is additionally reversed by the secretion of myokines from skeletal muscles during exercise, which can suppress the growth of breast cancer cells and encourage apoptosis [200]. Moreover, research has indicated that exercise can change gene expression associated with tumor suppression, including an increase in THSD7B, which is associated with decreased tumor growth and invasion [201].

The associations between metabolic syndrome and increased breast cancer risk are multifaceted and involve hormonal, inflammatory, and metabolic pathways. Metabolic syndrome, characterized by conditions such as obesity, hypertension, and insulin resistance, has been linked to poorer breast cancer outcomes and increased recurrence rates [202, 203].

Excess body weight results in increased estrogen production from adipose tissue, potentially promoting the growth of breast cancer cells. In tumors from overweight and obese women, increased levels of estrogen and progesterone receptors are observed, suggesting that a hormonal pathway is involved in tumor formation [204]. Additionally, adipose tissue in obese people releases proinflammatory cytokines that can foster a tumor‐promoting microenvironment [205].

This research yielded contradictory findings concerning coffee and tea intake, potentially shaped by the study design, differences in the populations examined, and the quantities of coffee and tea consumed. Although certain studies suggest that caffeine might increase the risk of breast cancer through its influence on estrogen metabolism, other studies propose that its high concentration of bioactive compounds, especially chlorogenic acids, could play a vital role in lowering oxidative stress and cancer risk due to their potent antioxidant and anti‐inflammatory effects [206].

The dietary regimen considerably affects breast cancer risk through several mechanisms, such as hormonal control, inflammation, and epigenetic modifications. Studies have shown that consuming many red and processed meats, along with fatty and sugary foods, is linked to a higher incidence of BC, whereas plant‐based diets provide protective advantages. The subsequent sections provide details on these mechanisms [207, 208].

The ways in which HRT and OCP are related to increased breast cancer risk include hormonal interactions and the dynamics of tumor growth. Studies have shown that progestins, rather than estrogens, are the main contributors to this increased risk, especially in the context of combined HRT. Research indicates that progestins increase the risk of breast cancer by stimulating progesterone receptor signaling, which may increase tumor development [209]. HRT promotes the development of current tumors, resulting in an increased occurrence of symptomatic breast cancer [210].

Breastfeeding is linked to a reduced risk of breast cancer via several biological and hormonal processes. The benefits of breastfeeding are complex and include alterations in mammary stem cells, shifts in hormone levels, and immune reactions that together lead to a reduced occurrence of breast cancer. Elevated levels of prolactin and oxytocin while breastfeeding encourage apoptosis in cells that may be cancerous. Lactation results in decreased exposure to progesterone and estrogen, lowering the risk of hormone receptor–positive breast cancers [211, 212]. Moreover, breastfeeding improves immune surveillance and regulates inflammation, potentially aiding in the removal of damaged cells and decreasing carcinogenesis [211].

Breast cancer occurs as a result of complex interactions between modifiable and nonmodifiable risk factors. Addressing these risk factors through comprehensive strategies, including lifestyle modifications such as improving one′s diet, engaging in regular physical activity, avoiding smoking/passive smoking, limiting alcohol consumption, maintaining a healthy weight after menopause, optimizing long‐term nonhormonal fertility choices, avoiding medically unnecessary hormone increases, and performing genetic screening when necessary, can help in the prevention of breast cancer. Bilateral prophylactic mastectomy is a recognized preventive measure for controlling breast cancer risk in individuals at high risk. Studies indicate that this surgical method lowers breast cancer rates by at least 90% in individuals with a strong family history [213]. Public health campaigns can focus on educating women about these risks and promoting healthier lifestyle choices to reduce the likelihood of developing breast cancer.

Understanding the different risk factors linked to breast cancer is essential for enhancing public health efforts, refining clinical care methods, and influencing effective healthcare policies. This information not only gives individuals the power to make educated choices regarding their health but also allows healthcare providers and policymakers to create focused approaches for prevention, early identification, and treatment, ultimately improving care quality for those at risk.

The study′s dependence on English‐language publications and limited time frame might have limited the findings by omitting important information. Furthermore, a quality evaluation of the articles was not carried out. Although this systematic review was conducted with a rigorous methodology to minimize bias, some potential biases in different steps, such as study selection, data extraction, and reporting, may still have influenced the results. In addition, differences in study design (prospective vs. retrospective), follow‐up time, confounding factors, participant count within the studies, and population differences can cause different results. Several risk factors remain underexplored. Future investigations should focus on factors related to chemical exposure, air pollution, stress and trauma, the gut microbiome, autoimmune diseases, viral infections, and epigenetics.

## 5. Conclusion

Lifestyle modifications such as improving one′s diet, engaging in regular physical activity, avoiding smoking/passive smoking, limiting alcohol consumption, maintaining a healthy weight after menopause, optimizing long‐term nonhormonal fertility choices, avoiding medically unnecessary hormone increases, and performing genetic screening when necessary can help in the prevention of breast cancer.

## Author Contributions

Z.M.: conceptualization, data curation, formal analysis, investigation, methodology, project administration, supervision, validation, visualization, writing—original draft, and writing—review and editing. Z.S.: data curation, investigation, methodology, writing—original draft, and writing—review and editing. L.A.: data curation, methodology, project administration, writing—original draft, and writing—review and editing. H.S.: conceptualization, investigation, methodology, project administration, resources, software, supervision, validation, visualization, writing—original draft, and writing—review and editing.

## Funding

No funding was received for this manuscript.

## Ethics Statement

The authors have nothing to report.

## Consent

The authors have nothing to report.

## Conflicts of Interest

The authors declare no conflicts of interest.

## Data Availability

Data sharing is not applicable to this article as no datasets were generated or analyzed during the current study.
